# Carta ao Editor

**DOI:** 10.1590/0034-7167.2022750602c

**Published:** 2022-05-06

**Authors:** Marina Peduzzi

**Affiliations:** I Universidade de São Paulo. São Paulo, São Paulo, Brasil.

Ilma. Sra.

Profa. Dra. Dulce Aparecida Barbosa, Editora-Chefe Revista Brasileira de Enfermagem

Vimos por meio desta apresentar a Vs. Sra. nossa solicitação de réplica ao artigo publicado recentemente na Revista Brasileira de Enfermagem de autoria de Hellen Emília Peruzzo e colaboradores^(1)^, visto que, no nosso entendimento, a publicação apresenta equívocos conceituais e metodológicos, tanto no tratamento do objeto de estudo, como no protocolo do estudo a luz de seu objetivo.

Nosso contato com Vs. Sra. se deve sobretudo a nossa preocupação em contribuir no adensamento do conhecimento sobre o tema ‘trabalho em equipe’ que é relevante no campo da Enfermagem e da Saúde, bem como evitar que mal-entendidos conceituais e metodológicos se perpetuem.

O estudo de Peruzzo et al^(1)^ propõe, no objetivo apresentado na p. 756: medir o clima organizacional no trabalho de profissionais das equipes da Estratégia Saúde da Família. No entanto utiliza a escala de Clima na Equipe, denominada na versão original de Anderson e West^(2)^ Team Climate Inventory, que constitui instrumento de medida de clima de equipe ou de grupos proximais de trabalho como referem os autores da escala^(1)^ (p. 757). Os próprios autores da escala argumentam que uma das razões para que esta produza altos níveis de consenso entre os membros da equipe se deve ao foco no próprio grupo proximal de trabalho, ao invés do foco na organização^(2)^(p. 254).

A concepção de clima utilizada na elaboração da escala original se refere a percepção compartilhada entre os membros da equipe^(2)^. O clima organizacional no plano das organizações se refere as “percepções e significados compartilhados sobre políticas, práticas e procedimentos que trabalhadores de uma organização vivenciam”^(3)^.

Cabe destacar que a dissertação de mestrado que validou a versão em português da escala Team Climate Inventory^(4)^ citado no artigo de Peruzzo, et al^(1)^ descreve que se trata de instrumento de medida de clima de equipe, bem como o artigo Silva, et al^(5)^.

Outro aspecto que causa estranheza no artigo de Peruzzo, et al^(1)^, p.757, é que as autoras descrevem que: “a análise não considerou cada equipe como unidade a ser avaliada, mas sim, o indivíduo em sua singularidade. Ou seja, o estudo publicado propõe medir clima organizacional, utiliza escala de medida de clima de equipe e recorta como unidade de analise o indivíduo (profissional).

No nosso entender, o estudo de Peruzzo, et al^(1)^ não contempla as diferenças conceituais entre clima organizacional e clima de equipe, tratando-os como sinônimos e também não considera as diferenças entre os termos: multiprofissional, interprofissional e interdisciplinar que segundo a literatura devem ser diferenciados pois o rigor na definição de termos e conceitos permitirá avanços no conhecimento sobre o tema.

Quanto aos equívocos referentes ao método de análise estatística, p. 757, o mais relevante é que a escala de Clima de Equipe é aplicada individualmente (profissionais da ESF no artigo referido) mas os escores médios devem ser analisados por agrupamento, ou seja, por equipes, visto que a escala mede a percepção compartilhada entre os membros da equipe sobre políticas, práticas e procedimento adotados pela equipe e não a percepção individual de cada profissional.

Por fim, reiteramos nossa solicitação de réplica ao artigo publicado por Peruzzo, et al^(1)^ considerando, como foi assinalado acima, evitar em especial que o equívoco conceitual e metodológico de equivalência entre clima organizacional e clima de equipe seja reproduzido em estudos futuros sobre trabalho em equipe em Enfermagem e em Saúde. Alternativamente a réplica, que não está prevista nas Normas da revista pensamos na possibilidade de encaminhamento de texto na modalidade de ‘reflexão’ que está previsto para submissão de manuscrito de “análise de diferentes pontos de vista, teóricos e/ou práticos”.

Colocamo-nos a disposição para qualquer esclarecimento necessário e subscrevemo-nos.

## References

[1] Peruzzo HE, Silva ES, Batista VC, Haddad MCFL, Peres AM, Marcon SS (2019). Organizational climate and teamwork at the Family Health
Strategy. Rev Bras Enferm.

[2] Anderson NR, West MA (1998). Measuring climate for work group innovation: development and
validation of the team climate inventory. J Organiz Behav.

[3] Schneider B, Ehrhart MG, Macey WH (2013). Organizational climate and culture. Ann Rev Psychol.

[4] Silva MC, Peduzzi M, Sangaleti CT, Silva D, Agreli HF, West MA (2016). Cross-cultural adaptation and validation of the teamwork climate
scale. Rev Saude Publica.

[5] Silva MC (2014). Adaptação transcultural e validação de instrumento de avaliação de
trabalho em equipe: Team Climate Inventory no contexto da Atenção Primária a
Saúde no Brasil.

