# Supplementary material for: Carta ao Editor
Source: Rev Bras Enferm. 2022 May 6;75(6):e2022750602c. [Article in Portuguese] doi: 10.1590/0034-7167.2022750602c (PMC9728877; doi:10.1590/0034-7167.2022750602c)
Supplement: Related texts

# Reply

**DOI:** 10.1590/0034-7167.2022750602cc

**Authors:** Dulce Barbosa

O Conselho Editorial da REBEn determinou a publicação da Carta ao Editor sem a resposta dos autores por falta de manifestação dos mesmos.

Atenciosamente, Dulce Barbosa

Editora Chefe da Revista Brasileira de Enfermagem
